# Task-Induced Functional Connectivity of the Syntax-Related Networks for Patients with a Cortical Glioma

**DOI:** 10.1093/texcom/tgaa061

**Published:** 2020-09-01

**Authors:** Kyohei Tanaka, Ryuta Kinno, Yoshihiro Muragaki, Takashi Maruyama, Kuniyoshi L Sakai

**Affiliations:** Department of Basic Science, Graduate School of Arts and Sciences, The University of Tokyo, Tokyo 153-8902, Japan; Department of Basic Science, Graduate School of Arts and Sciences, The University of Tokyo, Tokyo 153-8902, Japan; Division of Neurology, Department of Internal Medicine, Showa University Northern Yokohama Hospital, Yokohama 224-8503, Japan; Department of Neurosurgery, Tokyo Women’s Medical University, Tokyo 162-8666, Japan; Department of Neurosurgery, Tokyo Women’s Medical University, Tokyo 162-8666, Japan; Department of Basic Science, Graduate School of Arts and Sciences, The University of Tokyo, Tokyo 153-8902, Japan

**Keywords:** functional connectivity, frontal cortex, glioma, language, MRI

## Abstract

Analysis of the functional connectivity has enabled understanding of the cortical networks. In the present study, we used a picture-sentence matching task to introduce syntactically harder conditions, and clarified 3 major points. First, patients with a glioma in the lateral premotor cortex/inferior frontal gyrus or in other cortical regions showed much weaker activations than controls, especially in the left inferior frontal gyrus. Moreover, the error rates under the harder conditions were much higher for these patients. Secondly, syntactic loads induced selective connectivity with enhancement and suppression, consistently for both patients and controls. More specifically, the local connectivity was enhanced among the 3 syntax-related networks within the left frontal cortex, while the global connectivity of both dorsal and ventral pathways was suppressed. In addition, the exact reproducibility of *r*-values across the control and patient groups was remarkable, since under easier conditions alone, connectivity patterns for the patients were completely unmatched with those for the controls. Thirdly, we found an additional syntax-related network, further confirming the intergroup similarity of task-induced functional connectivity. These results indicate that functional connectivity of agrammatic patients is mostly preserved regardless of a glioma, and that the connectivity can change dynamically and systematically according to syntactic loads.

## Introduction

To characterize functional relationships among multiple cortical regions, assuming that they are all anatomically connected, one key concept would be functional connectivity. Functional connectivity has been defined as “the temporal correlation of a neurophysiological index measured in different brain areas” (Friston et al. 1993), and it can be applied to neuroimaging data such as positron emission tomography (PET) and functional magnetic resonance imaging (fMRI). For instance, a previous PET study on dyslexics clarified the importance of the functional connectivity between the left angular gyrus and occipital/temporal lobe for single-word reading abilities (Horwitz et al. 1998), and an fMRI study on normal participants (or healthy controls) reported enhanced connectivity between the Broca’s area and Wernicke’s area while they were listening to narrative texts (Hampson et al. 2002). In our previous fMRI studies, we found that functional connectivity among 14 cortical regions could be clearly divided into 3 groups of syntax-related networks (Kinno et al. 2014). Network I consisted of the left opercular/triangular parts of the inferior frontal gyrus (L. F3op/F3t), left intraparietal sulcus (L. IPS), right lateral premotor cortex (R. LPMC), R. F3op/F3t, presupplementary motor area (pre-SMA), and right posterior superior/middle temporal gyri (R. pSTG/MTG); Network II consisted of the L. LPMC, left angular gyrus (L. AG), lingual gyrus (LG), and cerebellar nuclei; and Network III consisted of the L. F3t, left orbital part of the F3 (L. F3O), L. pSTG/MTG, and left posterior middle/inferior temporal gyri (L. pMTG/ITG). Moreover, agrammatic patients, who had a glioma in either the left LPMC or F3, showed “chaotic” connectivity among the 14 regions (Kinno et al. 2015). There were at least 2 possible reasons for the connectivity changes we observed: (1) the presence of the glioma itself, and (2) the higher syntactic loads experienced by agrammatic patients. We later reanalyzed the functional connectivity data, and found an unexpected similarity of connectivity patterns among the patient groups (see the Results section), which could not be explained by the first possibility alone. Therefore, we hypothesize that the second possibility would significantly contribute to the connectivity changes. In the present study, we added 3 task conditions that required higher syntactic loads, and further examined associated changes in functional connectivity of the syntax-related networks.

**Figure 1:**
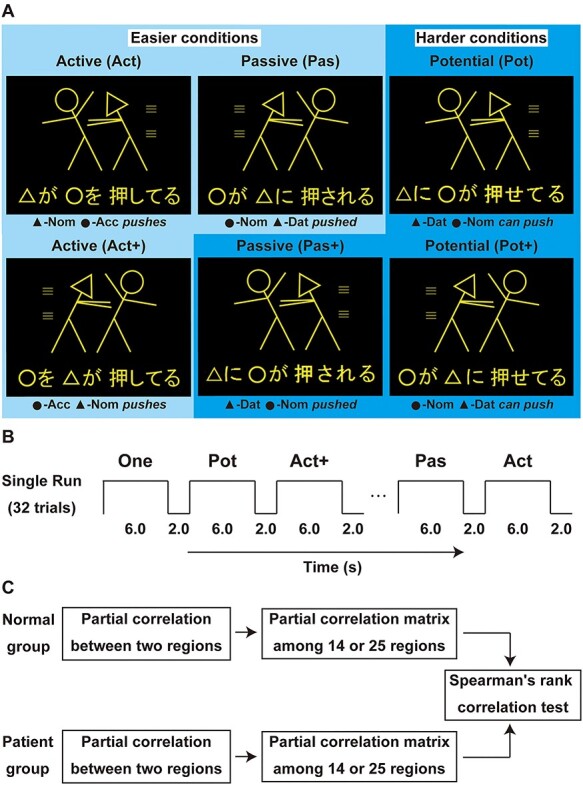
An experimental paradigm of the present study. (*A*) A picture-sentence matching task. Each stimulus consisted of 1 picture (top) and 1 sentence (bottom). Pictures consisted of 2 stick figures, each of which was distinguished by 1 of 3 “head” symbols: a circle, square or triangle. For sentence stimuli, we used 4 kinds of grammatical particles, which represent the syntactic information in Japanese: -*ga*, a nominative case marker; −*ni*, a dative case marker; −*o*, an accusative case marker; and -*to*, a coordinator (*and*). The 3 types of sentences in lighter blue represented the same conditions as in the previous study (Kinno et al. 2014): active (Act), passive (Pas), and scrambled active (Act+) sentences. Here, “+” denotes “with object scrambling,” making an object-initial sentence. Examples of matched sentences are shown here; for examples of unmatched sentences, see Fig. 1*A* in Kinno et al. (2014). In this study, we further introduced 3 additional types of sentences—the scrambled passive (Pas+), potential (Pot), and scrambled potential (Pot+) sentences—which are shown in darker blue. English translations of each sentence are as follows: “∆ *pushes* ○” (Act), “*As for* ○, ∆ *pushes it*” (Act+), “○ *is affected by* ∆‘*s pushing it*” (Pas), “*As for* ∆‘*s pushing*, ○ *is affected*” (Pas+), “∆ *can push* ○” (Pot), and “*As for* ○, ∆ *can push it*” (Pot+). (*B*) A typical run with task trials for MR scanning. (*C*) A flowchart of functional data analyses for normal and patient groups. Spearman’s rank correlation tests were performed between patient groups as well (see Fig. 2). Easier conditions = active (Act), passive (Pas), and scrambled active (Act+) conditions; harder conditions = scrambled passive (Pas+), potential (Pot), and scrambled potential (Pot+) conditions; One = one-argument condition.

In our previous studies (Kinno et al. 2008, 2009), we used a picture-sentence matching task, in which 3 types of Japanese sentences were tested (Fig. 1*A*, in lighter blue): active (abbreviated here as Act) (e.g., “○-*ga* □-*o oshiteru*,” “○ *pushes* □” in English), passive (Pas) (e.g., “□-*ga* ○-*ni osareru*,” “□ *is affected by* ○‘*s pushing it*”), and scrambled active sentences (e.g., “□-*o* ○-*ga oshiteru*,” “*As for* □, ○ *pushes it*”). Here, the term “scrambled” refers to “object scrambling,” in which an object to be emphasized is moved to the initial position of a sentence; the resultant scrambled sentence is perfectly grammatical in Japanese. The sentences with object scrambling are marked here with the symbol “+”; for example, scrambled active sentences are abbreviated as Act+. In our subsequent fMRI study (Tanaka et al. 2017), we further introduced 3 more types of sentences (Fig. 1*A*, in darker blue): scrambled passive (Pas+) (e.g., “○-*ni* □-*ga osareru*,” “*As for* ○‘*s pushing*, □ *is affected*”), potential (Pot) (e.g., “○-*ni* □-*ga oseteru*,” “○ *can push* □”), and scrambled potential (Pot+) sentences (e.g., “□-*ga* ○-*ni oseteru*,” “*As for* □, ○ *can push it*”). We have previously shown that the Pas and Act+ conditions were equally harder than the Act condition for some patients [see Fig. 1*D* in Kinno et al. (2014)], consistent with predicted syntactic loads [see Fig. 6*B* in Ohta et al. (2013)]. In combination with both of these effects, Pas + has larger loads than Act, Act+, or Pas; in addition, Pot and Pot+ have the largest loads among all of the conditions. We therefore called the Act, Act+, and Pas conditions “*easier* conditions,” and the Pas+, Pot, and Pot+ conditions “*harder* conditions.” Here, we analyzed the functional connectivity among all of 25 activated regions (Tanaka et al. 2017), including the same 14 regions mentioned above.

In the present study, we newly recruited patients with a glioma, and had them perform the same task under fully mixed easier and harder conditions, with the aim of comparing brain activations under both conditions, as we have previously reported (Tanaka et al. 2017), especially for those patients. In the tumor region, normal function can be well preserved because of infiltration rather than destruction (Ojemann et al. 1996; Krainik et al. 2003). Indeed, we have reported apparently normal left LPMC activations within the tumor region (Kinno et al. 2014), which could be a result of compensatory overactivation of remaining cells. We thus included the tumor region for the whole-brain analyses in the present study as well. We then examined any changes in functional connectivity due to higher syntactic loads. Our new data clearly indicate not only more variable and widespread functional connectivity, but also selective connectivity changes with both enhancement and suppression in a highly deterministic manner. Moreover, the functional connectivity was consistent and reproducible among the participant groups, irrespective of the presence of a glioma. It is intriguing that certain, as-yet-unknown laws regularize the activation in syntax-related networks in accordance with specific syntactic loads.

## Materials and Methods

### Participants

**Figure 2:**
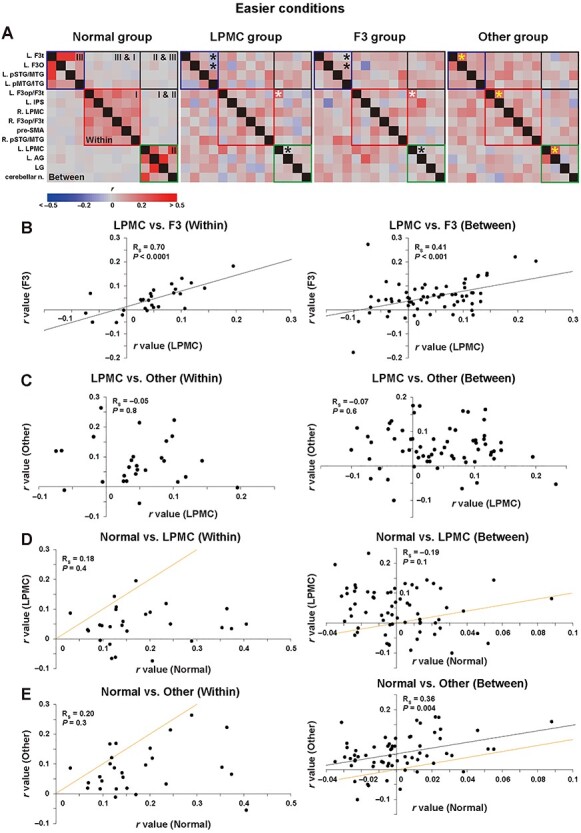
The unexpected similarity of the functional connectivity under the easier conditions for the LPMC and F3 groups. (*A*) The partial correlation matrices for the normal, LPMC, F3, and other groups (see the definition of each group in Results section) among the 14 regions reported previously (Kinno et al. 2015); for present purposes, the *r*-values were not converted into *Z*-values. In each matrix, the 3 syntax-related networks outlined in red, green, and blue correspond to networks I, II, and III, respectively. Here, “Within networks” refers to partial correlations between a pair of regions within individual networks (I, II, and III), while “*Between* networks” refers to those spanning 2 networks, that is, “cross-talks” between 2 networks (I & II, II & III, and III & I). For an explanation of the asterisks, see the main text. Note the unexpected similarity of the connectivity patterns for the LPMC and F3 groups. (*B–E*) Dot plot graphs of partial correlation coefficients shown separately for within or between networks. Each dot represents the partial correlation coefficient (*r*) between 2 of the 14 regions. Note the highly significant correlation (*R_S_*) of *r*-values for the LPMC and F3 groups (*B*), which was totally absent for the LPMC and other groups (*C*). Although there was no significant correlation for the normal and LPMC groups (*D*, both within and between), there was a significant correlation for the normal and other groups (*E*, between only). The lines in orange (*D–E*) denote diagonal or equivalent lines for *r*-values between the normal and compared groups. L = left; R = right; AG = angular gyrus; F3op/F3t/F3O = opercular/triangular/orbital parts of the inferior frontal gyrus; IPS = intraparietal sulcus; LG = lingual gyrus; LPMC = lateral premotor cortex; n. = nuclei; pMTG/ITG = posterior middle/inferior temporal gyri; pre-SMA = presupplementary motor area; pSTG/MTG = posterior superior/middle temporal gyri.

For the present study (except the reanalysis shown in Fig. 2), we recruited 38 patients in total, who were native Japanese speakers newly diagnosed as having a glioma. The patients preoperatively performed the picture-sentence matching task under the easier and harder conditions (Fig. 1*A*) during functional MRI scans at the University of Tokyo, Komaba. All but 1 patient (Table 1, Patient 13) then underwent surgery at the Department of Neurosurgery, Tokyo Women’s Medical University. The following conditions comprised the criteria for inclusion of these patients: (i) right-handedness, (ii) no deficits in verbal/written communication or other cognitive abilities reported by the patients or physicians, (iii) no history of neurological or psychiatric disorders other than glioma and seizures, (iv) freedom from seizures with or without antiepileptic drugs, (v) no medical problems related to MRI acquisition, (vi) completion of at least 3 fMRI runs without significant head movement, and (vii) an error rate of less than 20% under the control task (see the Stimuli and Tasks). Regarding criteria (i) and (vii), 6 and 2 participants were dropped, respectively.

**Table 1:** Demographics of each patient

Patient	Laterality quotient	Nonverbal IQ	Tumor location	Tumor volume (cm^3^)	Gray matter ratio (%)	Tumor type	Tumor grade
**LPMC/F3 group**							
Patient 1	100	124	L. F3op/F3t/F3O/OFG/STG/MTG/ITG	78.7	77.0	AOA	III
Patient 2	100	93	L. LPMC/F3op/F3t/F3O/STG/MTG/ITG/PrCG/PoCG/hippocampus	107.1	80.4	AA	III
Patient 3	100	118	L. F1/F2/SMA/LPMC/F3op/PoCG	101.3	64.9	AOA	III
Patient 4	80	109	L. F2/F3op/F3t/F3O/OFG/STG/cingulate	111.3	78.1	AO	III
Patient 5	78	109	L. F2/LPMC/F3op/F3t/F3O/STG	47.6	84.6	OL	II
Patient 6	100	124	L. LPMC/F3op/STG	8.3	88.7	DA	II
Patient 7	100	124	L. F2/F3t/F3O	20.8	74.8	DA	II
Patient 8	100	116	L. F1/F2/LPMC/PoCG	24.6	65.9	AA	III
Patient 9	100	124	L. F3t/F3O/STG	11.5	79.6	OL	II
Mean ± SD	95 ± 9	115 ± 11		56.8 ± 43.0	77.1 ± 7.8		
**Extra group**							
Patient 10	100	117	L. MTG/ITG/Hippo/FG	39.4	81.5	OL	II
Patient 11	60	98	L. ITG/FG/cingulate	18.8	80.5	AA	III
Patient 12	67	104	L. SMA/PreCG	20.9	70.1	AA	III
Patient 13	100	117	R. F3op/F3t	2.4	56.3	−	−
Patient 14	100	93	L. STG/MTG/ITG/AG/SMG/FG	32.6	84.8	AOA	III
Patient 15	89	122	L. STG/AG/SMG	28.0	80.7	AOA	III
Patient 16	100	124	L. MTG/ITG	4.7	87.3	OL	II
Patient 17	100	124	R. ITG/FG	4.9	94.0	AA	III
Patient 18	100	107	R. STG/MTG/ITG	26.4	81.3	AA	III
Mean ± SD	91 ± 16	112 ± 12		19.8 ± 13.3	79.6 ± 11		

Note: The laterality quotient of handedness was determined by the Edinburgh Handedness Inventory (Oldfield 1971). The nonverbal intelligence quotient was assessed with the Japanese version of the Wechsler Adult Intelligence Scale-III (2006; Harcourt Assessment Inc., San Antonio, TX). MR images were normalized with SPM12 to determine the tumor location and volume (cm^3^), as well as the gray matter ratio (%) of a tumor having both gray matter and white matter. The determination of tumor types and grades (II or III, with III being more severe) was based on the World Health Organization Classification of Tumors of the Nervous System (2016).

AA = anaplastic astrocytoma (grade III); AG = angular gyrus; AO = anaplastic oligodendroglioma (grade III); AOA = anaplastic oligoastrocytoma (grade III); DA = diffuse astrocytoma (grade II); F1 = superior frontal gyrus; F2 = middle frontal gyrus; F3O = orbital part of the F3; F3op = opercular part of the F3; F3t = triangular part of the F3; FG = fusiform gyrus; IQ = intelligence quotient; ITG = inferior temporal gyrus; L. = left; LPMC = lateral premotor cortex; MTG = middle temporal gyrus; OFG = orbitofrontal cortex; OL = oligodendroglioma (grade II); PrCG = precentral gyrus; PoCG = postcentral gyrus; SD = standard deviation; SMA = supplementary motor area; SMG = supramarginal gyrus; STG = superior temporal gyrus.

In order to clarify the contribution of the higher syntactic loads for the functional connectivity change, as explained in Introduction, we used the potential conditions that were grammatically difficult in the following points. The grammatical relation of a noun with -*ga* is the direct object in a potential sentence, while it is usually the subject in an active sentence. Moreover, the grammatical relation of a noun with -*ni* becomes the subject in a potential sentence, while it is the indirect object in a passive sentence [see Table 2 in Tanaka et al. (2017)]. Probably due to these facts, 6 of 22 normal participants in our previous study had error rates of more than 30% under the Pot or Pot+ condition, and thus they were dropped (Tanaka et al. 2017). In the present study, there were 10 patients out of 30 with error rates of more than 70% under the Pot and/or Pot+ conditions; because this rate was nearly equal to the frequency of the normal participants with high error rates, we dropped those 10 patients from analyses.

To separately examine the effect of a glioma in the left LPMC/F3 and that in other regions, 2 patients with a glioma extended widely from the left frontal to the left temporal/parietal regions (for more details on this criterion, see the next section) were excluded. The remaining 18 patients (listed in Table 1) were then divided into 2 groups (for the definition of groups, see the next section): *LPMC/F3* group [5 males and 4 females, aged 27–60 years, 43 ± 10 (mean ± standard deviation)] and Extra group (4 males and 5 females, aged 35–58 years, 48 ± 7.6). As the age was not normally distributed (Shapiro–Wilk test, *P <* 0.001), we used a nonparametric test to confirm the absence of significant difference in age (Mann–Whitney *U* test, *P* = 0.2), as well as in gender (Fisher’s exact test, *P* = 1). Controls, that is, normal group (12 males and 4 females, aged 20–40 years, 27 ± 6.6), consisted of the same participants as in our previous study (Tanaka et al. 2017); they were gender-matched with the patients (*P* = 0.2), but were younger than the patients (*P <* 0.0001). Regarding the detailed information for the reanalyzed participants and for the lesion identification methods, please see our previous papers Kinno et al. (2014) and Tanaka et al. (2017).

The categorization criterion of the patient groups was whether the cortical glioma of a patient did (LPMC/F3 group) or did not (Extra group) at least partially overlap on a voxel-by-voxel basis with the single cluster consisting of the L. LPMC, L. F3op/F3t, and L. F3O [see Fig. 3*B* in Tanaka et al. (2017)]. Regarding the 2 excluded patients with a widely extended glioma, more than 75% of their gliomas fell outside of the 15 left frontal regions, as defined by the Anatomical Automatic Labeling system (http://www.gin.cnrs.fr/en/tools/aal/) (Tzourio-Mazoyer et al. 2002). Lesion overlap maps were computed and visualized using the MRIcroN software (http://www.mccauslandcenter.sc.edu/mricro/mricron/).

This study involving human participants was reviewed and approved by the institutional review board of The University of Tokyo, Komaba, as well as of the Tokyo Women’s Medical University. The participants provided their written informed consent to participate in this study (No. 185–2 and 185–3).

### Stimuli and Tasks

Each visual stimulus consisted of a picture with head symbols (○, □, or ∆) at the top, and of an always grammatical sentence at the bottom (Fig. 1*A*). For each stimulus, we chose 2 different head symbols, and a sentence describing an action was written using a combination of the hiragana and kanji writing systems. We used 4 kinds of grammatical particles, which represent the syntactic information in Japanese: -*ga*, −*ni*, −*o*, and -*to* [a coordinator (*and*)]. Two sets of Japanese verbs (6 transitive verbs: *pull*, *push*, *scold*, *kick*, *hit*, and *call*; and 6 intransitive verbs: *lie*, *stand*, *walk*, *run*, *tumble*, and *cry*) were used [see Table 1 in (Tanaka et al. 2017)], each of which had either 4 or 5 syllables, controlling the numbers of syllables and letters among all conditions. Each sentence under the two-argument conditions (i.e., Act, Act+, Pas, Pas+, Pot, and Pot+, with 24 different stimuli each) had 2 arguments and ended with a transitive verb, while each sentence under the one-argument condition was a double-subjects (double-agents) type and ended with an intransitive verb. Half of the pictures depicted actions occurring from left to right, and the other half depicted actions occurring from right to left (see Fig. 1*A*); head symbols were also counterbalanced for both sides.

Each stimulus was presented visually in yellow against a dark background (Fig. 1*A*), and was presented for 6-s (fixed intratrial interval) followed by a 2-s blank interval (Fig. 1*B*). To minimize the effect of general memory demands, a whole sentence of a minimal length (i.e., 2 noun phrases and a verb) was visually presented for an ample time for the participants to respond. For fixation, a red cross was also shown at the center of the screen to minimize eye movements. The stimulus presentation and collection of behavioral data [error rates and response times (RTs)] were controlled using the LabVIEW software and interface (National Instruments, Austin, TX). The participants wore earplugs and an eyeglass-like MRI-compatible display (resolution, 800 × 600; VisuaStim Digital, Resonance Technology Inc., Northridge, CA).

In the picture-sentence matching task (Fig. 1*A*), the participants read a sentence silently and judged whether or not the action depicted in a picture matched the meaning of the sentence. They were instructed to respond by pressing 1 of 2 buttons in a row. Under the two-argument conditions, all mismatched sentences were made by exchanging 2 symbols in the original sentences, for example, “□ *pushes* ○” instead of “○ *pushes* □.” Under the one-argument condition, both symbol-mismatched sentences and action-mismatched ones were presented equally often, requiring the sentences to be read completely. The participants underwent short practice sessions before the task sessions.

For the Control (Cont) task, using the same stimulus sets of pictures and letters presented under the conditions described above, the participants judged whether or not the 2 head symbols in the picture matched those at the bottom, irrespective of their order. The letters in hiragana were jumbled without changing the head symbols and kanji, so that the letter string prevented even basic word recognition. A single run of the task sessions (256 s) contained 32 trial events (4 for each of the Act, Act+, Pas, Pas+, Pot, Pot+, one-argument, and Cont task conditions), the order of which was pseudorandomized to prevent any condition-specific strategy (Fig. 1*B*). Half of the stimuli consisted of matched picture-sentence pairs. 6 runs were administered, and the participants did not encounter the same sentence twice.

### MRI Data Acquisition and Analyses

The MRI scans were conducted on a 3.0 T system (GE Signa HDxt 3.0 T; GE Healthcare, Milwaukee, WI). We scanned 30 horizontal slices, each 3-mm thick and having a 0.5-mm gap, covering the range of *z* = −38.5 to 66 mm from the anterior to posterior commissure (AC–PC) line in the vertical direction, using a gradient-echo echo-planar imaging (EPI) sequence (repetition time (TR) = 2 s, echo time (TE) = 30 ms, flip angle (FA) = 90°, field of view (FOV) = 192 × 192 mm^2^, resolution = 3 × 3 mm^2^). In a single run, we obtained 128 volumes following 4 dummy images, which allowed for the rise of the MR signals. After completion of the fMRI runs, high-resolution T1-weighted images of the whole brain (136 axial slices, 1.0 × 1.0 × 1.0 mm^3^) were acquired from all participants (TR = 8.4 ms, TE = 2.6 ms, FA = 25°, FOV = 256 × 256 mm^2^).

The fMRI data were analyzed in a standard manner using SPM12 statistical parametric mapping software (Wellcome Trust Center for Neuroimaging, http://www.fil.ion.ucl.ac.uk/spm/) (Friston et al. 1995) implemented on MATLAB (Math Works, Natick, MA). We confirmed that all available fMRI data were free from large head movements, with a translation of <2 mm in the 3 directions and with a rotation of <1.4° around the 3 axes. The acquisition timing of each slice was corrected using the middle slice (the 15th slice chronologically) as a reference for the EPI data. The realigned data were resliced using seventh-degree B-spline interpolation, so that each voxel of each functional image matched that of the first volume.

Each participant’s T1-weighted structural image was coregistered to the mean functional image generated during realignment, bias-corrected with light regularization, and segmented by using default tissue probability maps and the Segment tool in the SPM12, which uses an affine regularization to warp images to the International Consortium for Brain Mapping Asian brain template (Ashburner and Friston 2005). The realigned functional images were also spatially normalized to the standard brain space as defined by the Montreal Neurological Institute (MNI), which converted voxel sizes to 3 × 3 × 3 mm^3^ and smoothed the images with an isotropic Gaussian kernel of 9-mm full-width at half maximum. Low-frequency noise was removed by high-pass filtering at 1/128 Hz.

In a first-level analysis (i.e., the fixed-effects analysis), each participant’s hemodynamic responses in each task session were modeled with a boxcar function (convolved with a hemodynamic response function) with a duration of 6 s from the onset of each visual stimulus. Only the functional data for trials with correct responses were modeled. For the technical reasons related to the presentation of the stimuli, a single run was removed from 2 participants. To minimize the effects of head movement, the 6 realignment parameters obtained from preprocessing were included as a nuisance factor in a general linear model. The images under each of the two-argument conditions, minus those under the one-argument condition, were then generated in the general linear model for each participant and used for the intersubject, across-subject comparison in a second-level analysis (i.e., the random-effects analysis). The functional data were thresholded at uncorrected *P <* 0.001 for the voxel level, and at corrected *P <* 0.05 for the cluster level, with family-wise error (FWE) correction across the whole brain. For the anatomical identification of activated regions, we basically used the Anatomical Automatic Labeling system and the labeled data as provided by Neuromorphometrics Inc. (http://www.neuromorphometrics.com/) under academic subscription.

### Functional Connectivity Analyses

By using the time-series data of each group, functional connectivity among multiple regions was assessed by a partial correlation method to estimate the direct connections for a pair of regions (though not their directionalities) (Smith 2012). Using a MarsBaR-toolbox (http://marsbar.sourceforge.net/), the time-series data were first averaged within a sphere of 6-mm radius centered at the local maximum of each region. To discount the global differences of signal changes among the runs, the time-series were normalized for each run. From each of the time-series of the 2 regions in question, we regressed out all the other nodes, before estimating the correlation between the two. For each participant, partial correlation coefficients (*r*) for each pair of regions were calculated using MATLAB, and they were averaged among all the participants to create a partial correlation matrix. For the correlation of the functional connectivity between 2 groups, we performed Spearman’s rank correlation tests and presented the results as Spearman’s rank correlation coefficients (*R_S_*), because *r*-values follow a non-Gaussian data distribution (Smith 2012) (see Fig. 1*C* for a flowchart of data analyses). Regarding the reanalysis shown in Figure 2, we used the data of partial correlation coefficients reported by Kinno et al. (2014), and performed Spearman’s rank correlation tests in the same manner as the analyses of the present data.

## Results

### The Unexpected Similarity of the Functional Connectivity Under the Easier Conditions for the LPMC and F3 Groups

Functional connectivity can be represented by partial correlation matrices, using partial correlation coefficients (*r*). Figure 2*A* shows those matrices for any pair of the 14 regions under the easier conditions, based on our previous papers (Kinno et al. 2014, 2015). The matrices are shown for the following normal and patient groups (28 participants in total, 7 for each group): normal group, LPMC group (patients with a glioma in the left LPMC), F3 group (patients with a glioma in the left opercular/triangular parts of the left F3), and other group (patients with a glioma in the other left frontal regions). As we have reported previously, the patients in the LPMC and F3 groups showed agrammatic comprehension, while the normal and other groups had normal syntactic comprehension [see Fig. 1*C*–*F* in Kinno et al. (2014)]. Regarding networks I–III (see Introduction), “Within networks” refers to partial correlations between a pair of regions within individual networks, while “Between” networks’ refers to those spanning 2 networks, that is, “cross-talks” between 2 networks. Note the clearly specific functional connectivity within each of the 3 syntax-related networks in the normal brain, as shown by their schematic [see Fig. 7*D* in Kinno et al. (2014)].

In reference to the normal group, the yellow asterisks indicate preserved connectivity of within networks for the other group (*r* > 0.20 for this group), whereas the black asterisks denote weaker connectivity of within networks for the LPMC and F3 groups. Based on this indication, since publishing the above-mentioned papers, we have noticed an unexpected similarity of connectivity patterns (i.e., 2D patterns of *r*-values) for the LPMC and F3 groups, not only for within networks, but also for between networks hitherto regarded as instances of “chaotic” connectivity. Indeed, the connectivity patterns were almost identical (see Fig. 2*A*), even for “noisy” partial correlations around *r* = 0 in both positive and negative values. It is also notable that stronger connectivity of the L. F3op/F3t-L. LPMC pair (with white asterisks) for between networks was consistent for the LPMC and F3 groups (*r* > 0.20 for both groups), which was absent for the normal or other group.

Given these intriguing findings, we directly compared the 2 matrices for the LPMC and F3 groups, by calculating the Spearman’s rank correlation coefficients (*R_S_*) among the *r*-values, separately for within and between networks (Fig. 2*B*). We observed significant correlations among *r*-values (within: *R_S_* = 0.70, *n* = 27, *P <* 0.0001; between: *R_S_* = 0.41, *n* = 64, *P <* 0.0001), irrespective of their different glioma locations and activation patterns [see Fig. 1*B*, 5*B*, and 5*G* in Kinno et al. (2014)]. This robust consistency between the 2 groups for the connectivity indicates that the number of participants was just adequate for the analyses. In contrast, there was no significant correlation for the LPMC and other groups (within: *R_S_* = −0.05, *P* = 0.8; between: *R_S_* = −0.07, *P* = 0.6) (Fig. 2*C*) or for the normal and LPMC groups (within: *R_S_* = 0.18, *P* = 0.4; between: *R_S_* = −0.19, *P* = 0.1) (Fig. 2*D*). The latter results were replicated for the normal and F3 groups as well (within: *R_S_* = 0.04, *P* = 0.8; between: *R_S_* = −0.14, *P* = 0.2).

On the other hand, regarding the comparison for the normal and other groups, the correlation was significant for between networks (*R_S_* = 0.36, *P* = 0.004), suggesting similar connectivity of overall cross-talks, although there was no correlation for within networks (*R_S_* = 0.20, *P* = 0.3) (Fig. 2*E*). Moreover, in reference to diagonal or equivalent lines shown in orange (for *r*-values between the normal and compared groups), the *r*-values from the patient groups were generally smaller for within networks, and larger for between networks (Fig. 2*C*,*D*), both indicating more noisy partial correlations regarding the 3 syntax-related networks. The LPMC and F3 groups thus had distinct and similar functional connectivity, which was completely different from that of the normal and other groups. This similarity cannot be explained by the presence of a glioma alone, because the glioma locations were clearly distinct among the patient groups, that is, the LPMC, F3, and other groups.

### Larger Syntactic Loads Under the Harder Conditions

**Figure 3:**
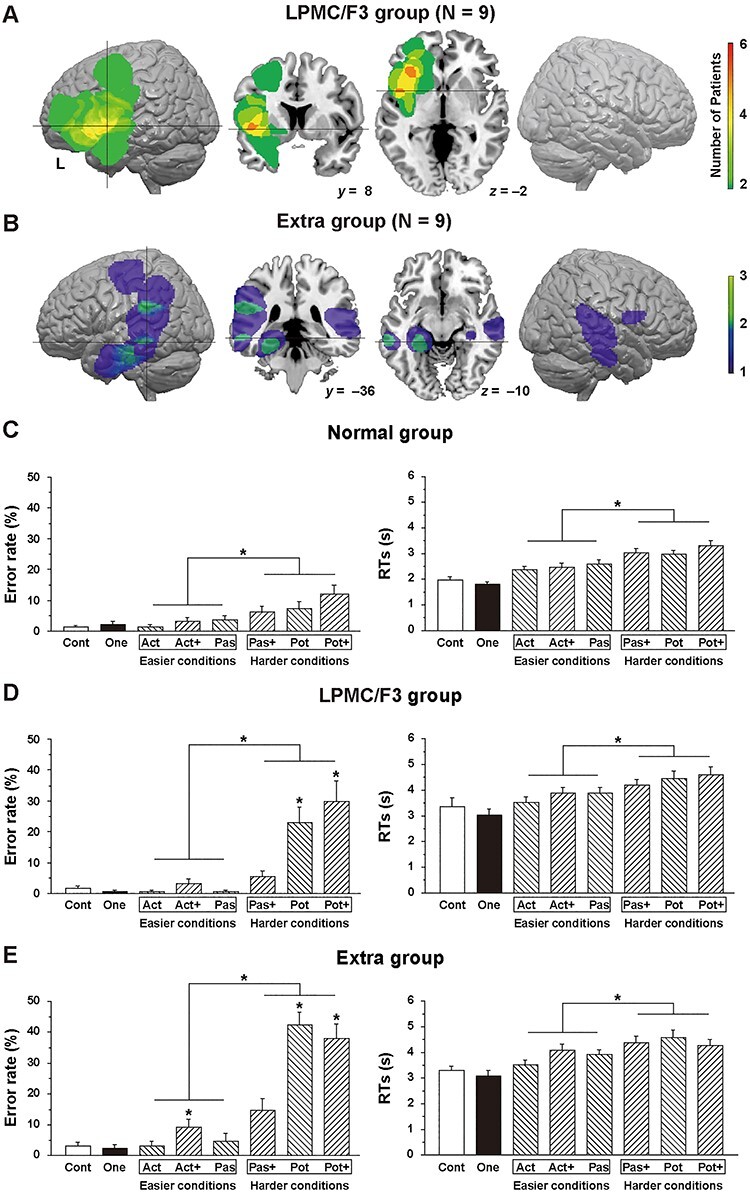
Lesion overlap maps and behavioral data. (*A*) Lesion overlap maps for the LPMC/F3 group. Lateral views and cross-sections (vertical cross hairs at *y* = 8, horizontal cross hairs at *z* = −2) of the standard brain are shown. The color scale denotes the number of patients. (*B*) Lesion overlap maps for the extra group. Lateral views and cross-sections (vertical cross hairs at *y* = −36, horizontal cross hairs at *z* = −10) of the standard brain are shown. (*C–E*) Histograms of error rates and RTs for the normal (*C*), the LPMC/F3 (*D*), and the extra groups (*E*). Error bars indicate the standard error of the mean for the participants. For all groups, both error rates and RTs under the harder conditions (Pas+, Pot, and Pot+; see Fig. 1A) were significantly larger than those under the easier conditions (Act, Act+, and Pas; *P < 0.001, paired *t*-test). An asterisk just above a bar denotes an error rates significantly higher than that under the same condition for the normal group (*P < 0.05, *t*-test). Cont = control task; One = one-argument condition; easier conditions = active (Act), passive (Pas), scrambled active (Act+) conditions; harder conditions = scrambled passive (Pas+), potential (Pot), scrambled potential (Pot+) conditions.

In the present study, the newly recruited patients were first characterized anatomically and behaviorally (Fig. 3). Lesion overlap maps showed that the LPMC/F3 group had a glioma mainly in the left F3 region (Fig. 3*A*), whereas most members of the extra group had a parietal or temporal lesion in a hemisphere (Fig. 3*B*; see Table 1 for detailed information). By introducing harder conditions, general cognitive loads might be additionally included in the task. However, the effect of general memory demands was minimized (see the Methods section), and behavioral data showed condition-dependent effects reflecting syntactic loads (Fig. 3*C*–*E*). A paired *t*-test showed higher error rates and longer RTs between the harder and easier conditions for the normal group [error rates: *t*(15) = 4.1, *P <* 0.001; RTs: *t*(15) = 9.0, *P <* 0.0001], which were replicated for the LPMC/F3 [error rates: *t*(8) = 4.4, *P <* 0.005; RTs: *t*(8) = 7.3, *P <* 0.0001] and extra [error rates: *t*(8) = 10, *P <* 0.0001; RTs: *t*(8) = 3.5, *P <* 0.01] groups. An unpaired *t*-test showed that, when compared with the error rates of the normal group, the error rates under the Pot and Pot+ conditions were higher for the LPMC/F3 group [Pot: *t*(23) = 3.1, *P <* 0.005; Pot+: *t*(23) = 2.8, *P <* 0.01], and those under the Act+, Pot, and Pot+ conditions were higher for the extra group [Act+: *t*(23) = 2.3, *P <* 0.05; Pot: *t*(23) = 8.0, *P <* 0.0001; Pot+: *t*(23) = 5.1, *P <* 0.0001)]. These results confirmed the presence of syntactic loads for all of the 3 groups tested here.

### Distinct Activation Patterns Among the Normal and Patient Groups

**Figure 4:**
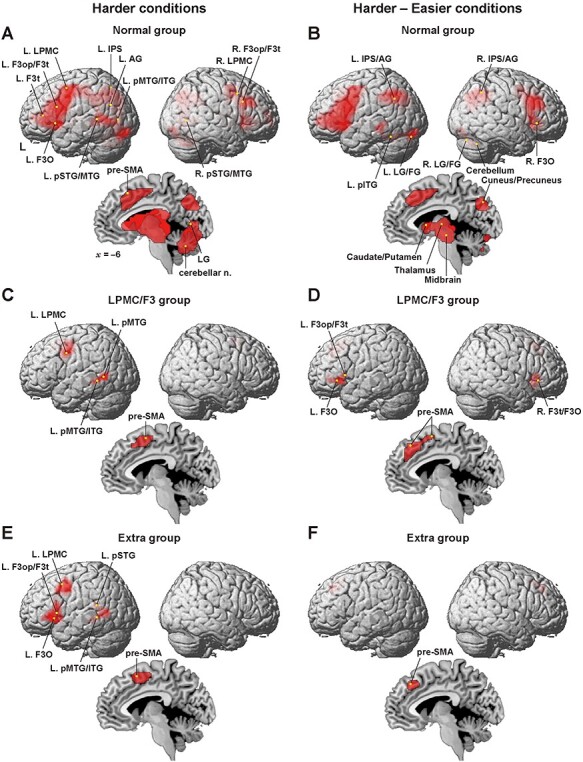
Distinct activation patterns among the normal and patient groups. (*A*) Significant regions identified by the harder conditions for the normal group. Activations were projected onto the left (L) and right lateral surfaces of a standard brain (FWE-corrected *P <* 0.05). A medial section is also shown. Yellow dots denote foci of the previously identified 14 regions of the 3 syntax-related networks (Table 2). (*B*) Significant regions identified by the contrast [harder–easier] for the normal group. Yellow dots denote 11 additional regions (see Table 2). (*C–F*) Significant regions for the LPMC/F3 (*C, D*) and extra (*E, F*) groups, identified by the harder conditions (*C, E*) and by the contrast [harder–easier] (*D, F*). Each yellow dot shows the local maximum of an activated region (see Table 3). In these group analyses, the full factorial option was used, except in the case of panel (*B*), where we used the flexible factorial option from our previous study [see Fig. 3A in Tanaka et al. (2017)]. L = left; R = right; AG = angular gyrus; F3op/F3t/F3O = opercular/triangular/orbital parts of the inferior frontal gyrus; IPS = intraparietal sulcus; FG = fusiform gyrus; LG = lingual gyrus; LPMC = lateral premotor cortex; n. = nuclei; pSTG/MTG/ITG = posterior superior/middle/inferior temporal gyri; pre-SMA = presupplementary motor area.

All of the 14 regions reported previously as the 3 syntax-related networks (see Introduction) were activated under the harder conditions for the normal group (Fig. 4*A*). Moreover, 11 additional regions (see Table 2) were further localized by the contrast [harder–easier] (Fig. 4*B*), as reported previously (Tanaka et al. 2017). The present study further revealed dramatically reduced activation for both the LPMC/F3 and extra groups (Fig. 4*C*–*F*, Table 3). Regarding the LPMC/F3 group, activation under the harder conditions was localized in the L. LPMC, L. pMTG/ITG, and pre-SMA (Fig. 4*C*); the reduction was particularly prominent in the left F3, which corresponded to the lesion site (see Fig. 3*A*). Moreover, activation by the contrast [harder–easier] was restricted in the ventral portion of the bilateral F3, as well as in the pre-SMA (Fig. 4*D*). We confirmed that activation in the L. F3op/F3t, L. F3O, and R. F3t/F3O was below the threshold under the harder conditions.

**Table 2:** Previously reported regions for the normal group

Network	Brain region	BA	Side	*x*	*y*	*z*
I	F3op/F3t	44/45	L	–45	18	27
	IPS	7/39/40	L	−21	−72	51
	*IPS/AG	7/39	L	−33	−58	35
	*IPS/AG	7/39	R	36	−58	41
	LPMC	6/8	R	30	3	45
	F3op/F3t	44/45	R	33	18	24
	pre-SMA	6/8	M	9	24	51
	pSTG/MTG	22/21	R	60	−57	3
II	LPMC	6/8	L	−48	3	42
	AG	39	L	−33	−60	18
	LG	18	M	−3	−69	6
	Cerebellar nuclei		M	−3	−51	−27
	*Cerebellum		R	27	−64	−34
	*Midbrain		M	−6	−19	−13
III	F3t	45	L	−48	33	6
	F3O	47	L	−36	15	−6
	*F3O	47	R	30	29	−1
	pSTG/MTG	22/21	L	−57	−48	0
	pMTG/ITG	37/19	L	−45	−69	0
IV	*pITG	37/19	L	−45	−49	−22
	*LG/FG	18/19	L	−24	−85	−19
	*LG/FG	18/19	R	24	−85	−13
	*Cuneus/Precuneus	7/18/19	M	−6	−76	35
	*Caudate/Putamen		L	−15	8	5
	*Thalamus		M	−9	−16	5

Note: Stereotactic coordinates (*x*, *y*, *z*) in the Montreal Neurological Institute space are shown for each activation peak of *Z*-values. The threshold was set at corrected *P <* 0.05 for the cluster level. The asterisks (*) denote 11 additionally regions under the harder–easier conditions in the normal group (Tanaka et al. 2017), whereas the other 14 regions have been previously reported as networks I–III (Kinno et al. 2014).

BA = Brodmann’s area; L = left; M = medial; R = right; p = posterior; AG = angular gyrus; F3op/F3t/F3O = opercular/triangular/orbital parts of the inferior frontal gyrus; FG = fusiform gyrus; IPS = intraparietal sulcus; LPMC = lateral premotor cortex; LG = lingual gyrus; STG/MTG/ITG = superior/middle/inferior temporal gyrus; pre-SMA = presupplementary motor area.

**Table 3:** Activated regions in the LPMC/F3 and extra groups

Brain region	BA	Side	*x*	*y*	*z*	Z
**LPMC/F3 group**						
Harder conditions						
LPMC	6/8	L	−33	2	35	5.0
pre-SMA	6/8	M	−6	5	59	5.3
pMTG/ITG	37/19	L	−51	−43	−4	4.2
pMTG	37	L	−48	−52	5	3.6
Harder–easier conditions						
F3t	45	L	−48	20	2	3.9
F3O	47	L	−36	29	−4	4.3
		L	−42	41	−4	4.1
F3t/F3O	45/47	R	36	29	−1	4.4
pre-SMA	6/8	M	−6	2	59	3.6
		M	−3	32	44	3.9
**Extra group**						
Harder conditions						
LPMC	6/8	L	−39	5	56	5.0
		L	−42	8	50	5.0
F3op/F3t	44/45	L	−51	20	8	4.3
F3t/F3O	45/47	L	−48	23	−1	5.3
pre-SMA	6/8	M	−3	−1	53	4.2
		M	−6	11	53	3.8
pSTG	22	L	−54	−40	17	3.7
		L	−45	−49	14	4.1
pMTG/ITG	37/19	L	−57	−58	−1	3.8
		L	−51	−43	−4	4.1
Harder–easier conditions						
pre-SMA	6/8	M	3	20	53	3.8
			−6	35	44	3.8

Note: Stereotactic coordinates (*x*, *y*, *z*) in the Montreal Neurological Institute space are shown for each activation peak of Z-values. The threshold was set at corrected *P <* 0.05 for the cluster level.

BA = Brodmann’s area; L = left; M = medial; R = right; p = posterior; F3op/F3t/F3O = opercular/triangular/orbital parts of the inferior frontal gyrus; LPMC = lateral premotor cortex; STG/MTG/ITG = superior/middle/inferior temporal gyrus; pre-SMA = presupplementary motor area.

In contrast in the extra group, activation under the harder conditions was localized in the left frontal cortex (the L. LPMC, L. F3op/F3t, and L. F3O), left temporal cortex (the L. pSTG and L. pMTG/ITG), and pre-SMA (Fig. 4*E*). This activation pattern should not be regarded as abnormal in spite of its significant reduction, because the left frontal and temporal activations were matched with those of the normal or other group under the easier conditions alone [see Fig. 5*A*,*D* in Kinno et al. (2014)]. Under the contrast [harder—easier], activation was observed only in the pre-SMA (Fig. 4*F*). These results indicate that activation patterns are crucially influenced by a glioma, depending on their locations.

### Reproducible Functional Connectivity Among the 14 Regions for the Normal and Patient Groups

**Figure 5:**
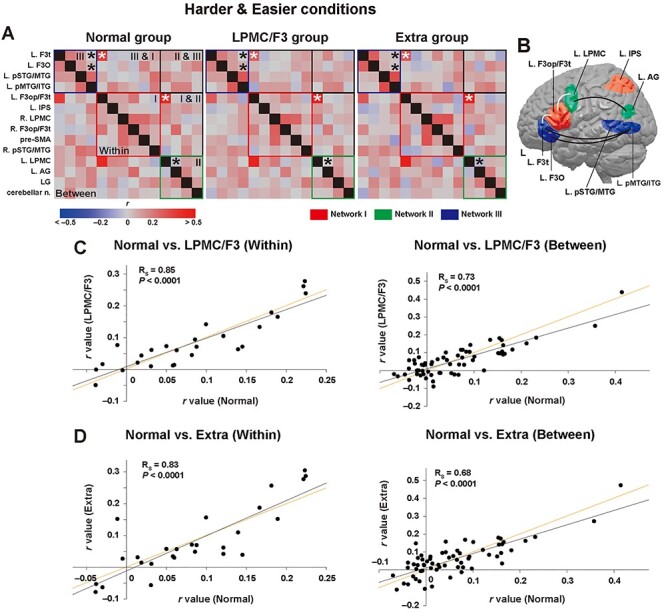
Reproducible functional connectivity among the 14 regions for the normal and patient groups. (*A*) Partial correlation matrices under the easier and harder conditions for the normal, LPMC/F3, and extra groups, presented as described in Figure 2. (*B*) The left brain regions for networks I–III are depicted in red, green, and blue, respectively. The white lines connecting 2 regions denote enhanced functional connectivity for between networks, which corresponds to the white asterisks in (*A*). The black lines denote suppressed functional connectivity for within networks, which corresponds to the black asterisks in (*A*). (*C, D*) Dot plot graphs of the partial correlation coefficients (*r*), separately shown for within and between networks. The lines in orange denote diagonal or equivalent lines, indicating that the *r*-values were precisely reproduced between the normal and patient groups. L = left; R = right; AG = angular gyrus; F3op/F3t/F3O = opercular/triangular/orbital parts of the inferior frontal gyrus; IPS = intraparietal sulcus; LG = lingual gyrus; LPMC = lateral premotor cortex; n. = nuclei; pMTG/ITG = posterior middle/inferior temporal gyri; pre-SMA = presupplementary motor area; pSTG/MTG = posterior superior/middle temporal gyri.

In spite of the marked differences in activation pattern among the normal, LPMC/F3, and extra groups (Fig. 4), the functional connectivity among the 14 regions was reproducible for the 3 groups (Fig. 5*A*). For example, consistency can be clearly observed in enhanced functional connectivity (white asterisks) for between networks (*r* > 0.20 for all the 3 groups), as well as in suppressed connectivity (black asterisks) for within networks. This reproducibility among the 3 groups for the connectivity indicates that the number of participants was just adequate for the analyses. This is a surprising result, if we note the absence of correlations among the normal, LPMC/F3, and other groups in the previous results (Fig. 2*C*–*E*). Moreover, these connectivity changes precisely matched those (except for the L. F3t-L. F3op/F3t pair) for the LPMC and F3 groups, as shown in Figure 2*A* (white and black asterisks). Figure 5*B* schematically shows the enhanced local connectivity among the 3 key regions of the left frontal cortex (white lines), as well as the suppressed global connectivity (black lines). The latter global connectivity is known as the dorsal and ventral pathways, that is, 2 major language-related tracts. Together, these results indicate the possibility that the harder conditions, that is, the higher syntactic loads, caused the selective connectivity changes, including both enhancement and suppression.

We then confirmed the intergroup similarity by the highly significant correlations for the normal and LPMC/F3 groups (within: *R_S_* = 0.87, *n* = 27, *P <* 0.0001; between: *R_S_* = 0.75, *n* = 64, *P <* 0.0001) (Fig. 5*C*), as well as for the normal and extra groups (within: R_S_ = 0.76, *P <* 0.0001; between: *R_S_* = 0.66, *P <* 0.0001) (Fig. 5*D*). Correlations were also significant for the LPMC/F3 and extra groups (within: *R_S_* = 0.88, *P <* 0.0001; between: *R_S_* = 0.64, *P <* 0.0001). Moreover, the regression lines shown in the 4 panels almost matched the diagonal or equivalent lines, indicating the exact reproducibility of *r*-values among all 3 groups. The regression lines for between networks were slightly less steep than the diagonal lines, indicating relatively lower *r*-values for the patient groups. These results demonstrate that the functional connectivity was independent of the existence of a glioma, but was reproducible and thus meaningful in a highly deterministic manner.

### Reproducible Functional Connectivity among the 25 Regions for the Normal and Patient Groups

**Figure 6:**
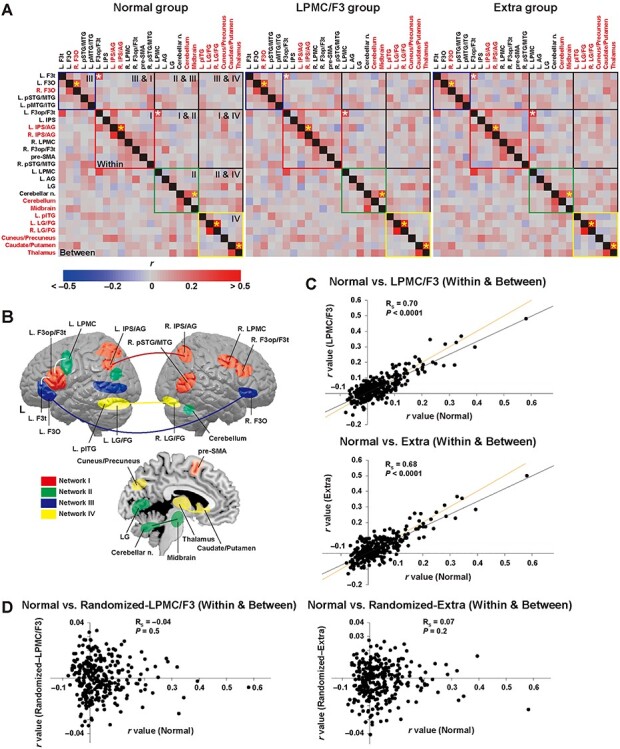
Reproducible functional connectivity among the 25 regions for the normal and patient groups. (*A*) Partial correlation matrices under both the easier and harder conditions for the normal, LPMC/F3, and extra groups. The 4 networks outlined in red, green, blue, and yellow correspond to networks I–IV, respectively. The yellow asterisks denote the stronger connectivity for within networks (*r* > 0.20 for all of the 3 groups); white asterisks are also shown and correspond to the connectivities shown in Figure 5A. (*B*) All of the 25 regions are shown for networks I–IV in red, green, blue, and yellow, respectively. The enhanced connectivity between 2 regions [yellow asterisks in (*A*)] is shown by colored lines: a red line for network I, a green line for network II, a blue line for network III, and yellow lines for network IV. (*C*) Dot plot graphs of the partial correlation coefficients, shown for both within and between networks. The orange lines indicate the exact reproducibility of *r*-values between the normal and patient groups. (*D*) Partial correlation coefficients calculated with the randomized time-series data in the LPMC/F3 or extra group, showing complete absence of correlations with the normal group. L = left; R = right; AG = angular gyrus; F3op/F3t/F3O = opercular/triangular/orbital parts of the inferior frontal gyrus; IPS = intraparietal sulcus; FG = fusiform gyrus; LG = lingual gyrus; LPMC = lateral premotor cortex; n. = nuclei; pSTG/MTG/ITG = posterior superior/middle/inferior temporal gyri; pre-SMA = presupplementary motor area.

We then incorporated the 11 additional regions (Fig. 4*B*) into the syntax-related networks, thereby examining functional connectivity among all of the 25 regions (Fig. 6*A*; additional region names in red). The R. F3O, bilateral IPS/AG, and cerebellum/midbrain were classified into networks III, I, and II, respectively, based on their bilateral connections (except LPMC) and anatomical proximity to the 14 previously identified regions. The other regions of the L. pITG, bilateral lingual/fusiform gyrus (LG/FG), cuneus/precuneus, caudate/putamen, and thalamus were newly assigned to “Network IV.” The specificity of network IV was confirmed by significantly larger *r*-values for within network IV than those for between networks (IV & I, IV & II, and IV & III), according to a Wilcoxon rank sum test (normal group, *W* = 256, *P <* 0.0001). Moreover, the sensitivity of network IV was shown by equally large *r*-values for within network IV and those for within networks I–III (normal group, *W* = 107, *P* = 0.4). The yellow asterisks denote the stronger connectivity for within networks (*r* > 0.20 for all 3 groups), which included all pairs of the bilateral regions indicated above. The emergence of network IV can be characterized by higher connectivity between the bilateral LG/FG, as well as between the caudate/putamen and thalamus in all 3 groups (Fig. 6*A*). Figure 6*B* schematically shows the enhanced connectivity between 2 regions for network IV, as well as for the other networks (colored lines representing the yellow asterisks in Fig. 6*A*). These results indicate that the higher syntactic loads induced the local and bilateral connectivity, with an additional contribution of network IV.

We also confirmed intergroup similarity both for the within and between networks (Fig. 6*C*). Correlations were highly significant for the normal and LPMC/F3 groups (*R_S_* = 0.70, *n* = 300, *P <* 0.0001), as well as for the normal and extra groups (*R_S_* = 0.68, *P <* 0.0001). Correlations were also significant for the LPMC/F3 and extra groups (*R_S_* = 0.65, *P <* 0.0001). Just as for the similarity shown in Figure 5*C*,*D*, the regression lines shown in the 2 panels matched the orange lines, indicating the exact reproducibility of *r*-values among all 3 groups. The regression lines were also slightly less steep than the diagonal lines, indicating lower *r*-values for the patient groups. These strong correlations among the 3 groups were not due to the larger sample size of region pairs, because the coefficients calculated with randomized time-series data in the LPMC/F3 or the extra group showed no significant correlation with those for the normal group (normal vs. randomized-LPMC/F3: *R_S_* = −0.04, *P* = 0.5; normal vs. randomized-extra: *R_S_* = 0.07, *P* = 0.2) (Fig. 6*D*). These results clearly demonstrate the existence of intergroup similarity among the 25 identified regions of the 4 syntax-related networks, which were found to be mostly functional even for the patients with a glioma.

## Discussion

Before discussing various points related to our study, we summarize 3 major findings. First, patients with a glioma in the LPMC/F3 regions or in other cortical regions (Fig. 3*A*,*B*) showed much weaker activations than controls, especially in the left F3 (Fig. 4), indicating that the activation patterns were affected by a glioma in various regions. Moreover, the error rates under the harder conditions (Fig. 1*A*) were much higher for these patients (Fig. 3*D*,*E*). Secondly, syntactic loads induced selective connectivity with enhancement and suppression, consistently for both patients and controls (Fig. 5*A*). More specifically, the local connectivity was enhanced among the 3 syntax-related networks (networks I–III) for the L. F3t-L. F3op/F3t and L. F3op/F3t-L. LPMC pairs, while the global connectivity of both dorsal and ventral pathways was suppressed (Fig. 5*B*). Moreover, the exact reproducibility of *r*-values among both the normal and patient groups (Fig. 5*C*,*D*) was remarkable, since under easier conditions alone, connectivity patterns for the patients were completely unmatched with those for the controls (Fig. 2*A*,*D*). Thirdly, we found an additional syntax-related network (network IV; Fig. 6*A*,*B*), further confirming the intergroup similarity of task-induced functional connectivity (Fig. 6*C*). These results indicate that the functional connectivity of agrammatic patients is mostly preserved in spite of the presence of a glioma, and that the connectivity can change dynamically and systematically according to syntactic loads.

The loss of functional connectivity for the LPMC and F3 groups shown in Figure 2*A* was previously considered to be “chaotic” (Kinno et al. 2015). The present study, however, revealed the similarity in the connectivity between these 2 groups (Fig. 2*B*), irrespective of differences in their glioma location and activation pattern. We also found that selective connectivity including enhancement and suppression was actually replicated by the normal group, when the harder conditions were additionally introduced (compare Fig. 2*A* and Fig. 5*A*). These results indicate that the modification of the connectivity for the LPMC and F3 groups was produced not by the existence of the glioma alone, but by a higher demand of syntactic loads for the agrammatic patients. This interpretation is supported by the activation pattern for the normal group, in that the main effect of sentence construction, that is, the [passive–active conditions] & [potential–passive conditions] contrast, which reflects specific syntactic loads, caused significant activation in the L. LPMC, L. F3op/F3t, and L. F3O, as well as in the pre-SMA [see Fig. 3*B* in Tanaka et al. (2017)]. Note that this activation pattern precisely matches that for the extra group (Fig. 4*E*). Because the local connectivity among networks I–III was enhanced in compensation for the suppression of the global connectivity in networks II and III, the syntax-related networks were functionally reorganized due to the introduction of syntactically harder conditions, further demonstrating the dynamic and deterministic nature of functional connectivity.

The suppressed global connectivity (Fig. 5*A*) corresponds to the dorsal and ventral pathways, which have been widely known for their roles supporting language functions (Hickok and Poeppel 2007; Friederici 2012). As shown in Figure 5*B* (black lines), the dorsal and ventral pathways belong to networks II and III, respectively. Among these pathways in each hemisphere, we have recently reported that the fractional anisotropy (FA) of the left arcuate fasciculus was significantly correlated with individual accuracy of a syntactic task in a second language (L2) (Yamamoto and Sakai 2016). Moreover, senior high-school students with higher performances in L2 showed significantly higher FA of the left arcuate fasciculus than other groups with lower performances, demonstrating the critical roles of this left dorsal pathway in language acquisition (Yamamoto and Sakai 2017). It is interesting to note that higher syntactic loads actually suppress this within network pathway, thereby utilizing the L. F3op/F3t of network I to combine networks II and III. In an fMRI study of Japanese Sign Language, we previously showed that activated regions in the left frontal cortex gradually expanded in the dorso-ventral axis, in correspondence with a difference in linguistic units in the following order: word-, sentence-, and discourse-levels (Inubushi and Sakai 2013). The highly connected local networks in the left frontal regions thus subserve the central units for processing syntactic structures at the linguistic levels and demands.

Our previous study showed that the functional connectivity for within networks was significantly higher than that for between networks in the normal and other groups, whereas there was no such difference for the LPMC or F3 group [see Fig. 1 in Kinno et al. (2015)]. Moreover, here we observed a similar connectivity for between networks in the normal and other groups (Fig. 2*E*). This result indicates that the connectivity for between networks was not random but meaningful during the syntactic task. Consistent with these findings, the connectivity for between networks was highly reproducible among the normal and patient groups (Fig. 5*C*,*D*). These results cannot be explained merely by the upregulation of intrinsically bursting neurons in the peritumoral zone (de Groot et al. 2012), and specific task requirements should be taken into account.

In the present study, 5 regions were additionally introduced into networks I–III, and 6 regions were newly classified into network IV. The R. F3O was added to network III and showed strong connectivity with the contralateral L. F3O (the blue line in Fig. 6*B*). Another bilateral pair of the L. IPS/AG and R. IPS/AG were also added to network I, which showed strong connectivity between them (the red line in Fig. 6*B*). These results indicate the importance of combining bilateral counterparts together as a syntax-related network. Moreover, the cerebellum and midbrain were added into network II, making enhanced connectivity with the cerebellar nuclei (the green line in Fig. 6*B*). The involvement of the cerebellum in higher cognitive functions such as thinking has been proposed (Ito 1993).

As shown in Figure 6*A*,*B*, network IV consisted of the L. pITG, bilateral LG/FG, cuneus/precuneus, caudate/putamen, and thalamus, and strong connectivity was observed between the bilateral LG/FG, as well as between the caudate/putamen and thalamus (the yellow lines in Fig. 6*B*). Among these regions, the precuneus has major subcortical connections with the dorsum of the thalamus, dorsolateral caudate nucleus, and putamen (Cavanna and Trimble 2006). It has been indicated that these subcortical structures are associated with spatial neglect in humans (Karnath et al. 2002), and thalamic functions are critical in attentional control and cognitive control in general (Halassa and Kastner 2017). Moreover, cognitive control/monitoring in language switching has been associated with the left caudate nucleus (Crinion et al. 2006), and a recent fMRI study of bilinguals reported that the bilateral LG/FG extending to the L. pITG was additionally activated together with the bilateral caudate (Ma et al. 2014). Multiple sclerosis patients with atrophy in the basal ganglia and thalamus also exhibited slower cognitive processing speed (Batista et al. 2012). Taking these pieces of evidence together, Network IV could be regarded as a regulation system for the higher-order cognitive functions, including syntactic processing.

In conclusion, higher syntactic loads under the harder conditions induced reproducible and meaningful functional connectivity not only for the patients with a glioma, but also for the controls in this study, thereby dynamically controlling the local and global pathways. The present results thus imply the validity of using specific tasks for patients with cognitive deficits during rehabilitation, since higher task loads would be expected to improve and retrieve specific functional connectivity in the brain, either locally or globally.

## Notes

We thank N. Komoro for technical assistance, and H. Matsuda for administrative assistance. *Conflict of Interest*: None declared.

## Funding

Grants-in-Aid for Scientific Research (B) (No. JP17H02347) and (C) (No. JP17K01978) from the Ministry of Education, Culture, Sports, Science, and Technology of Japan, and a grant from the Brain Science Foundation.
